# How Attention to Faces and Objects Changes Over Time in Toddlers with Autism Spectrum Disorders: Preliminary Evidence from An Eye Tracking Study

**DOI:** 10.3390/brainsci9120344

**Published:** 2019-11-27

**Authors:** Filippo Muratori, Lucia Billeci, Sara Calderoni, Maria Boncoddo, Caterina Lattarulo, Valeria Costanzo, Marco Turi, Costanza Colombi, Antonio Narzisi

**Affiliations:** 1 Department of Developmental Neuroscience, IRCCS Fondazione Stella Maris, 56128 Calambrone, PI, Italy; f.muratori@fsm.unipi.it (F.M.); vcostanzo@fsm.unipi.it (V.C.); anarzisi@fsm.unipi.it (A.N.); 2 Department of Clinical and Experimental Medicine, University of Pisa, 56128 Calambrone, PI, Italy; 3 Institute of Clinical Physiology, National Research Council of Italy (CNR), 56128 Calambrone, PI, Italy; lucia.billeci@ifc.cnr.it; 4 Programma Interdipartimentale “Autismo 0–90”, A.O.U. Policlinico G. Martino, 98124 Messina, ME, Italy; mariaboncoddo@gmail.com; 5 Stella Maris Mediterraneo Foundation, 85032 Chiaromonte, PZ, Italy; lattarulo@asmbasilicata.it (C.L.); turimarc@gmail.com (M.T.); 6 Department of Psychiatry, University of Michigan, Ann Arbor, MI, 48109, USA; ccolombi@med.umich.edu

**Keywords:** autism spectrum disorders, toddlers, eye tracking, joint attention, longitudinal

## Abstract

Further understanding of the longitudinal changes in visual pattern of toddlers with autism spectrum disorders (ASDs) is needed. We examined twelve 19 to 33-month-old toddlers at their first diagnosis (mean age: 25.1 months) and after six months (mean age: 31.7 months) during two initiating joint attention (IJA) tasks using eye tracking. Results were compared with the performance of age-matched typically developing (TD) toddlers evaluated at a single time-point. Autistic toddlers showed longitudinal changes in the visual sensory processing of the IJA tasks, approaching TD performance with an improvement in the ability to disengage and to explore the global space. Findings suggest the use of eye tracking technology as an objective, non-intrusive, adjunctive tool to measure outcomes in toddlers with ASD.

## 1. Introduction

Autism spectrum disorders (ASDs) are neurodevelopmental conditions, affecting approximately 1% of children in Italy [1], and characterized by persistent deficits in social communication and interaction, along with the presence of restrictive and repetitive behaviors [2]. Eye tracking is a technique that is opening new avenues for quantitative, objective, simple, non-invasive evaluation of the visual patterns in young individuals with ASD [3,4]. In particular, it can be used to explore ASD atypicalities in visual social attention [5], the behavior of allocating attentional resources to social stimuli [6], and an area in which deficits have been well documented in individuals with ASD [7,8,9]. A more advanced form of social attention is joint attention (JA), which is the ability to coordinate visual attention with another individual to an object or event that emerges between 6 and 12 months of age in typical development [10]. Two types of JA are described in the literature: (1) Response to joint attention (RJA), which is the ability to follow the direction of other’s gaze; and (2) initiating joint attention (IJA), which is the ability to use gaze to direct the attention of others towards a shared object or event of interest [11]. JA impairment is consistently reported as one of the earliest and specific signs of ASD that becomes apparent at the end of the first year of life [12,13,14,15]. 

Eye tracking is a method that enables high-precision detection and accuracy characterization of the subtle variations in the spontaneous viewing patterns of JA in individuals with ASD [16,17]. Since it does not require advanced motor responses or language skills, eye tracking can offer useful insights when studying infants and toddlers with ASD [18,19].

Through a previous eye tracking study, we analyzed RJA and IJA in toddlers with ASD [20]. Results indicated different visual patterns between ASD and typically developing (TD) toddlers in IJA only. Specifically, toddlers with ASD looked longer at faces and had more transitions from the target object to the face, while TD toddlers looked more at the non-target object, and had more transitions from the non-target object to the face or from one object to another. These counterintuitive findings have been discussed in relation to the impairment in disengagement from face and in divided attention, which might compromise the ability to track more than one object on the scene. 

On the basis of this previous investigation, in the current paper, we aimed to evaluate possible longitudinal changes of the visual pattern during the same IJA tasks in toddlers with ASD. To accomplish this aim, the same IJA tasks of our previous study were administered longitudinally, with an interval of six months. In particular, we focused on testing whether changes in the visual pattern of toddlers with ASD were following a developmental trajectory similar to that identified in typical development. Finally, we aimed to explore whether some clinical measures were predictive of visual pattern changes in toddlers with ASD.

## 2. Method

### 2.1. Participants

Twelve toddlers with ASD and 15 age- and gender-matched TD toddlers participated in the study (Table 1). The sample of toddlers with ASD only partially (six subjects) overlaps that of our previous study [20], while the sample of TD is the same. The clinical diagnosis of ASD was established according to DSM-5 criteria [2], and confirmed by using algorithm cutoffs on the Autism Diagnostic Observation Schedule (ADOS-2) [21]. Exclusion and inclusion criteria were presented elsewhere [18]. All children (ASD and TD) received a non-verbal developmental evaluation through the administration of the performance subscale of the Griffiths Mental Developmental Scales (GMDS) [22]. The adaptive behavior profile of children with ASD was measured by means of the Vineland-II, a semi-structured interview with the individual’s caregiver [23]. Control toddlers were typically developing according to parental report, and did not have any medical or developmental diagnoses. Typical development was also confirmed by the Child Behavior Check List 1.5–5 (CBCL) questionnaire [24]. All toddlers in the TD group scored below the borderline/clinical range (Table 1).

ADOS-2 items belonging to the joint attention factor (pointing, gesture, showing, initiation of joint attention, unusual eye contact) were chosen as measures of JA [25]. 

All parents provided written informed consent, including permission to use the video recordings for scientific reasons. The experimental procedures and the informed consent were approved by the Institutional Review Board of the Clinical Research Institute for Child and Adolescent Neurology and Psychiatry.

### 2.2. Procedure and Stimuli

Toddlers with ASD were assessed at their first diagnosis—time 1 (T1) (mean age, 25.1 months; SD, 4.6 months; age range, 19–33 months; and after six months—time 2 (T2) (mean age, 31.7 months; SD, 4.7 months). The comparison group of TD toddlers was assessed only at the first time point (T1: Mean age, 26.5 months; SD, 4.1 months; age range: 18–30 months).

Eye tracking data were acquired using an SMI-RED 500 Eye Tracker (SMI, SensoMotoric Instruments, Teltow, Germany). Both eyes were tracked with a rated accuracy < 1° and a sampling frequency of 120 Hz, which is a sufficient sampling frequency rate to detect two-point data according to an authoritative study [26]. The toddlers sat on a child chair, approximately 50 cm from the monitor, in front of a small table. No explicit instructions were given. The experiment started with a 5-point calibration sequence, in which a cartoon was used as calibration point to catch the toddlers’ attention to the screen. The calibration was repeated until the deviation from the known calibration target for both the x and y components was below 2°. 

**Figure 1:**
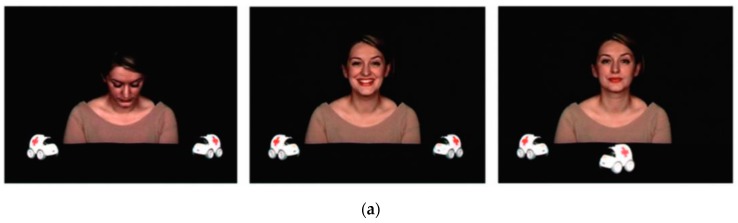

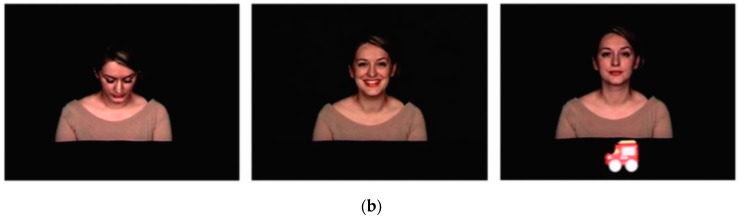
Initiating joint attention (IJA) task. (**a**) IJA task with a predictable event (IJA-1); (**b**) IJA task with an unpredictable event (IJA-2).

The IJA paradigm consisted of two different tasks: (1) IJA task with a predictable event (IJA-1): A female model was positioned between two little cars placed on the table in front of her, and one of the two cars (‘target object’) moved, while the actor maintained a direct gaze to the child with a neutral expression; (2) IJA with an unpredictable event (IJA-2): The same female actor was initially alone in the scene, and then a toy truck (“target object”) appeared unexpectedly from outside of the scene and crossed the screen while the actor maintained a direct gaze with a neutral expression. Each task included three phases: (a) Looking down (2 s); (b) smiling (2 s); (c) JA (7 s) (Figure 1 includes screenshot of the video). Four trials per conditions were presented to each child. Each trial was preceded by a colorful “attention-getter” that was displayed at the center of the screen until the toddler looked at it for at least 500 ms. The total duration of the eye tracking session was, on average, two min (the duration varied slightly from one child to another, according to their ability of performing the calibration and of looking at the attention-getter).

### 2.3. Data Analysis

Measures of JA were calculated on the JA segments of the tasks. Measures referred to transitions and were computed by extracting raw data and analyzing them in Matlab (MathWorks, Natick, MA, USA) using homemade scripts. Specifically, we evaluated the number of transitions from face to target object and the number of transitions from objects to face (as an indication of the alternating looking pattern between them). In IJA-1, we also computed between-object transitions and the normalized transition score (that is, the difference between the total number of transitions from target object to face and the total number of transitions from non-target object to face divided by the total number of transitions from either object to face). 

In addition, we selected the following areas of interest (AOIs): Model’s face, target object, and non-target object (the object that did not move in the IJA-1 task). For each of these AOIs, we calculated fixation duration (FD), computed as a percentage of the total (i.e., FD on that AOI relative to the participants’ on-trial FD). A fixation threshold of 60 ms was applied.

### 2.4. Statistical Analysis

Statistical analysis was completed using SPSS 20 software for Mac (SPSS, Chicago, IL, USA). Descriptive analyses for the continuous variables (means and standard deviations) and ordinal variables (frequencies and percentages) were performed on the demographic and clinical variables. Normality of the data was evaluated using the Shapiro–Wilk test and the equality of the variances with Mauchly’s sphericity test.

For the inferential analyses, three tests were performed: (1) One-way ANCOVAs to evaluate differences at T1 in the visual pattern between ASD toddlers and TD, using developmental level as a covariate; (2) a repeated measures ANCOVA (T1 versus T2) for ASD, to evaluate changes on clinical and eye tracking measures using the difference between age at T1 and age at T2 as a covariate; (3) one-way ANCOVAs to evaluate differences in the visual pattern between toddlers with ASD at T2 and the visual pattern of TD toddlers at T1, using developmental level and age as covariates. The significance threshold for all tests was set at 0.05 after Bonferroni correction. Effect sizes were estimated by partial eta squared (η^2^). 

A stepwise linear regression was performed to identify T1 clinical measures predicting eye tracking performance at T2. Associations between eye tracking and clinical measures at T2 were examined using Spearman’s correlations. In addition, in order to evaluate whether modifications in eye tracking pattern were associated with modifications in social functioning, we compared clinical measures (ADOS items measuring the “joint attention factor” [27] and Vineland-II items [23]) at T1 and T2, using paired-sample *t*-tests.

## 3. Results

Results of the eye tracking measures are reported in Table 2.

### 3.1. ASD and TD Comparison at T1

In IJA-1, toddlers with ASD had significantly higher transitions from target object to face (*p* = 0.026), and significantly higher normalized transition scores (*p* = 0.036) compared to TD. Conversely, TD toddlers made significantly higher transitions from non-target object to face (*p* = 0.029), and had higher fixations to the non-target object (*p* = 0.016) than toddlers with ASD. No other significant differences were detected. In the IJA-2 task, toddlers with ASD had both significant higher transitions from target object to face (*p* = 0.011) and from face to target object (*p* = 0.012) than TD toddlers. Moreover, toddlers with ASD had significant higher FD to face (*p* = 0.01).

### 3.2. Longitudinal Changes in ASD and Comparison with TD

**Figure 2:**
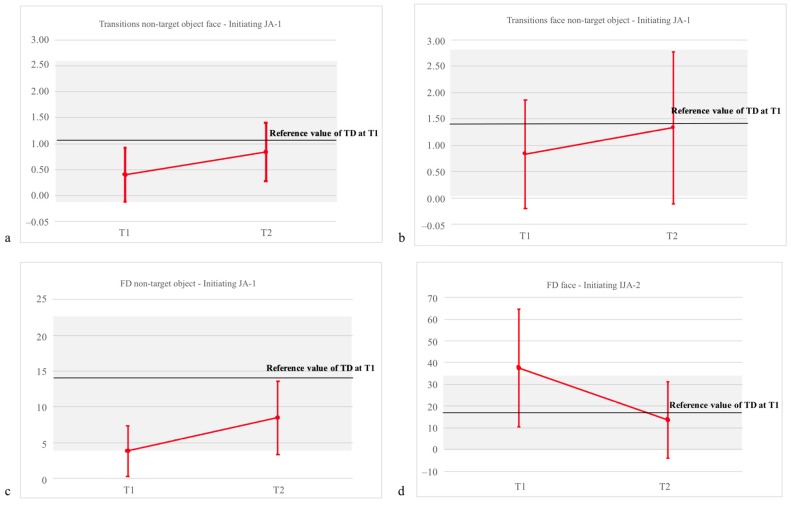
Significant longitudinal changes (with SD) in eye tracking measures in the ASD group. For the purpose of comparison, the reference values in the TD group are reported as a black line. (**a**) Change in transitions from non-target object to face in the Initiating JA-1 task; (**b**) change in transitions from face to non-target object in the Initiating JA-1 task; (**c**) change in fixation duration at non-target object in the Initiating JA-1 task; (**d**) change in fixation duration at face in the Initiating JA-2 task.

In the IJA-1 task, toddlers with ASD showed a significant increase in transitions from non-target object to face with time (*p* = 0.024), so that at T2, no significant difference was still present between ASD and TD (Figure 2a). In IJA-1, toddlers with ASD also showed a significant increase (*p* = 0.04) of transitions from face to non-target object (Figure 2b). In addition, there was a significant increase with time of FD to the non-target object (*p* = 0.038), so that at T2, no significant difference was still present between ASD and TD (Figure 2c).

In the IJA-2 task, no significant effect of time for transitions was observed, but a significant decrease with time of FD to face was noticed (*p* = 0.03), so that at T2, no significant difference between ASD and TD was detected as far as FD for face is regarded (Figure 2d). Pairwise comparison showed that while transitions from face to target object were still higher at T2 in ASD compared to TD (*p* = 0.04), differences in transitions from target object to face disappeared at T2.

### 3.3. ADOS Predictors of Eye Tracking Performance at T2

**Figure 3:**
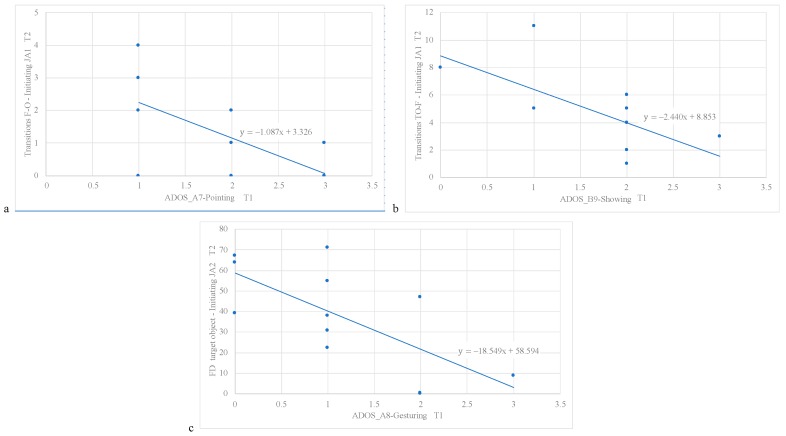
Significant clinical predictors at T1 for eye tracking measures at T2 in the ASD group. (**a**) ADOS_A7-Pointing at T1 as an independent predictor of transitions from face to non-target object at T2 in the IJA-1 task; (**b**) ADOS_B9-Showing at T1 as an independent predictor of transitions from target object to face at T2 in the IJA-1 task; (**c**) ADOS_A8-Gesturing at T1 as an independent predictor of fixation duration at target object at T2 in the IJA-2 task.

For the IJA-1 task, it was observed that ADOS_A7-Pointing at T1 was an independent predictor of transitions from face to non-target object at T2 (β = −0.63, adj-R^2^ = 0.34, *p* = 0.027) (Figure 3a), and that ADOS_B9-Showing at T1 was an independent predictor of transitions from target object to face at T2 (β = −0.64, adj-R^2^ = 0.35, *p* = 0.025) (Figure 3b).

Finally, for the IJA-2, it was observed that ADOS_A8-Gesturing at T1 was an independent predictor of FD at target object at T2 (β = −0.64, adj-R^2^ = 0.34, *p* = 0.035) (Figure 3c).

### 3.4. Correlations with Developmental Quotient

The Performance developmental quotient at T1 was not a predictor of any change in eye tracking measure at T2. No significant correlation between GMDS-Performance or difference in GMDS-Performance between T2 and T1 and eye tracking measures were found in ASD at T2.

### 3.5. Longitudinal Modifications in Clinical Measures

As regards ADOS items, ADOS-2_A8-Gesturing significantly changed from T1 to T2 (T1: 1.20 ± 0.91; T2: 0.50 ± 0.52; *p* = 0.038). 

As far as the Vineland-II scores, significant modifications in the items “Receptive” (T1: 12.00 ± 3.39; T2: 20.40 ± 8.90; *p* = 0.04), “Expressive” (T1: 14.00 ± 9.94; T2: 23.20 ± 10.94; *p* = 0.04), and “Community” (T1: 4.60 ± 3.97; T2: 7.60 ± 4.33; *p* = 0.039) were observed.

## 4. Discussion

While confirming that toddlers with ASD show an atypical visual pattern for IJA compared to toddlers with TD, the findings of the present investigation support the hypothesis of early longitudinal changes in the visual pattern of toddlers with ASD toward a greater similarity to that characteristic of TD subjects. Over a period of six months, the visual pattern of ASD is no longer characterized by the prevalence of fixation to face and by the indifference to non-target object. Moreover, significantly more transitions from non-target object to face are observed.

These three modifications make the visual performance of toddlers with ASD in both IJA tasks very different from their previous performance at T1, and more similar to the performance of TD toddlers six months younger. Two mutually reinforcing factors can be called into question. First, we can hypothesize that a maturational process of the anatomical systems supporting JA occurred [28,29,30]. 

Second, we have to mention that all toddlers with ASD in our sample are engaged in some type of behavioral and or psycho-educational intervention, which may boost neuroplasticity [31]. In fact, JA constitutes a primary target for early ASD intervention [32,33,34], which in turn may have had a positive impact on developmental trajectories of JA, as previously observed [35]. In this framework, a seminal randomized clinical trial detected that a group of ASD children that carried out a specific developmental behavioral intervention showed both neurotypical patterns of cortical activation and increased neural response to social stimuli [36]. Thus, based on our findings, we can speculate that IJA atypicalities detected in ASD toddlers at T1 represent delay rather than impairment, since they could improve over time. 

In a previous study with older children with ASD [37], using an integrating eye tracking and electroencephalography approach, we reported trends of changes in both brain activity and connectivity in the JA circuits after a six-month rehabilitative intervention, which were correlated with modifications in gaze measures. Thus, we can also hypothesize that the longitudinal modifications observed in the present study are associated with a modification of neurophysiological mechanisms.

The global longitudinal changes in the visual sensory processing of our IJA tasks seem to be linked to the increasing of transitions and, in particular, of transitions to non-target object. We suppose that this increase of transitions is an expression of the improved abilities in attention disengagement and in the global space exploration, which represent two skills typically impaired in early ASD. 

Indeed, a preference for local over global processing has been repeatedly indicated as a core feature of the autistic phenotype (e.g., [38,39]). Moreover, previous studies reported that infants later diagnosed with ASD were slower to disengage their attention from one object to another, compared to TD infants [18,40,41].

The increased ability of our toddlers with ASD to shift their attention from face to non-target object should therefore be interpreted as a positive sign for the development of IJA and, more broadly, social competencies [42]. Accordingly, the developmental changes in the visual sensory processing of the IJA tasks are related to improvements both in the social behaviors included in the “JA factor” of the ADOS-2 [27], and in specific items measuring Communication and Daily Living Skills of the Vineland-II [23]. This block of evidence makes the current eye tracking findings more robust from a translational point of view. Notably, the modifications we observed are independent from developmental quotient, i.e., they are not attributable to modifications in developmental skills from T1 to T2. Thus, repeated eye tracking evaluation may represent an objective and specific outcome measure in toddlers with ASD [43], since it is able to detect modifications in the visual pattern reflecting brain plasticity of the social brain. 

Finally, we examined clinical predictors of modifications in eye tracking profile among toddlers with ASD, and we observed that low clinical measures of autism severity at some ADOS-2 items (i.e., pointing, showing, and gestures) were correlated with increased eye tracking longitudinal changes. This negative correlation indicates that a less severe clinical performance on these measures at an earlier age can be a good predictor for the approaching of the IJA visual pattern to that of TD. Previous studies correlated eye tracking data with clinical outcome. For example, a recent cross-sectional study of infant siblings has demonstrated that less gaze alternations between an interaction partner and an interesting event at 10 months was associated with more social impairment and less showing and pointing at 18 months [44]. In addition, different visual responses to dynamic social stimuli in toddlers with ASD have been linked to differences in autism severity and developmental functioning 1–2 years later [45].

Several limitations need to be acknowledged in this study. First, as this was a small sample investigation, replication of the initial finding is needed. Second, since this study did not include the eye tracking measure at T2 for the TD group, it is not possible to compare the pattern of eye gaze between ASD and control subjects in the longitudinal evaluation. Consequently, it remains to be elucidated whether differences between groups are present at T2 also, and/or whether new differences emerge. Despite these drawbacks, this study suggests changes in looking patterns of toddlers with ASD during a brief interval (i.e., six months) that results in IJA performance more similar to those of subjects with TD at T1. Our data do not allow disentangling the relative contributions of rehabilitative treatment and normal brain maturation on changes in eye tracking profile. Future large-scale randomized controlled trials after standardized rehabilitative intervention are necessary before translating eye tracking evaluation into a treatment outcome measure to include in clinical practice.

## Figures and Tables

**Table 1:** Participants characteristics.

	ASD T1 *n* = 12	ASD T2 *n* = 12	TD *n* = 15	ASD T1 vs. ASD T2 *p*-Value	ASD T1 vs. TD T1 *p*-Value	ASD T2 vs. TD T1 *p*-Value
	M (SD)	M (SD)	M (SD)			
Age (months)	25.1 (4.6)	31.7 (4.7)	26.5 (4.1)	−	*t*(24) = 0.86, *p* = 0.40	−
Gender: M, F	10, 2	10, 2	13, 2	−	*χ*^2^ = 0.06, *p* = 0.81	−
ADOS-2, total	14.9 (4.5)	11.0 (3.7)	−	*t*(22) = −2.31, *p* = 0.03	−	−
GMDS, performance	74.9 (25.0)	83.5 (13.6)	102.5 (11.7)	*t*(11) = −1.73, *p* = 0.11	*t*(24) = 3.79, *p* = 0.001	*t*(24) = 3.88, *p* = 0.001
Vineland-II, total	75.8 (4.4)	79.6 (15.9)	−	*t*(4) = −0.86, *p* = 0.43	−	−

ASD: Autism spectrum disorders; TD: Typically developing; M: Mean; SD: Standard deviation; ADOS: Autism Diagnostic Observation Schedule; GMDS: Griffiths Mental Developmental Scales.

**Table 2:** Eye tracking measures.

	ASD T1 *n* = 12 Mean (SD)	ASD T2 *n* = 12 Mean (SD)	TD *n* = 14 Mean (SD)	ASD T1 vs. ASD T2 *p*-Value	ASD T1 vs. TD T1 *p*-Value	ASD T2 vs. TD T1 *p*-Value
Initiating JA-1						
FD: Face	24.85 (20.11)	19.34 (17.06)	20.14 (14.61)	F = 0.86, *p* = 0.36, η^2^ = 0.03	F = 0.53, *p* = 0.47, η^2^ = 0.02	F = 0.07, *p* = 0.79, η^2^ = 0.03
FD: Target object	22.15 (13.69)	34.54 (23.16)	38.62 (20.62)	F = 2.17, *p* = 0.17, η^2^ = 0.18	F = 2.22, *p* = 0.15, η^2^ = 0.08	F = 2.15, *p* = 0.16, η^2^ = 0.08
FD: NT object	3.84 (3.51)	8.48 (5.11)	13.56 (9.92)	F = 4.37, *p* = 0.038 *, η^2^ = 0.537	F = 6.69, *p* = 0.016 *, η^2^ = 0.218	F = 1.29, *p* = 0.27, η^2^ =0.05
T to face from target object	5.15 (4.11)	4.58 (2.87)	1.42 (1.13)	F = 0.89, *p* = 0.77, η^2^ = 0.01	F = 5.781, *p* = 0.026 *, η^2^ = 0.216	F = 2.48, *p* = 0.12, η^2^ = 0.09
T to face from NT object	0.40 (0.52)	0.84 (0.56)	1.38 (1.19)	F = 6.29, *p* = 0.024 *, η^2^ = 0.711	F = 5.55, *p* = 0.029 *, η^2^ = 0.217	F = 2.22, *p* = 0.18, η^2^ = 0.07
Normalized transition score	0.84 (0.20)	0.77 (0.27)	0.40 (0.48)	F = 0.80, *p* = 0.39, η^2^ = 0.08	F = 4.99, *p* = 0.036 *, η^2^ = 0.185	F = 3.49, *p* = 0.07, η^2^ = 0.132
T from face to target object	4.08 (2.27)	4.75 (2.73)	3.27 (2.08)	F = 1.08, *p* = 0.25, η^2^ = 0.13	F = 0.99, *p* = 0.33, η^2^ = 0.04	F = 0.64, *p* = 0.43, η^2^ = 0.03
T from face to NT object	0.83 (1.03)	1.33 (1.43)	1.33 (1.44)	F = 5.67, *p* = 0.04 *, η^2^ = 0.36	F = 1.07, *p* = 0.31, η^2^ = 0.04	F = 0.35, *p* = 0.56, η^2^ = 0.02
Between object transitions	3.89 (3.43)	4.42 (3.11)	5.07 (3.91)	F = 4.55, *p* = 0.06, η^2^ = 0.29	F = 0.21, *p* = 0.15, η^2^ = 0.08	F = 2.08, *p* = 0.16, η^2^ = 0.08
Initiating JA-2						
FD: Face	37.57 (27.03)	13.65 (17.52)	16.76 (16.86)	F = 6.00, *p* = 0.03 *, η^2^ = 0.375	F = 8.02, *p* = 0.01 *, η^2^ = 0.276	F = 0.001, *p* = 0.97, η^2^ = 0.0001
FD: Target object	28.52 (22.12)	34.54 (23.16)	31.84 (22.30)	F = 0.62, *p* = 0.45, η^2^ = 0.06	F = 0.07, *p* = 0.79, η^2^ = 0.003	F = 0.12, *p* = 0.73, η^2^ = 0.006
T from target object to face	4.42 (1.78)	4.00 (1.79)	1.71 (1.37)	F = 1.93, *p* = 0.19, η^2^ = 0.18	F = 7.65, *p* = 0.011 *, η^2^ = 0.242	F = 6.21, *p* = 0.15, η^2^ = 0.20
T from face to target object	4.0 (2.0)	4.79 (2.75)	1.31 (1.37)	F = 2.62, *p* = 0.14, η^2^ = 0.23	F = 7.47, *p* = 0.012 *, η^2^ = 0.237	F = 4.57, *p* = 0.04 *, η^2^ = 0.222

FD: fixation duration; T: transitions; NT: non-target object. * Significant at 0.05 after Bonferroni correction. All significant *p*-values are reported in bold.

## Data Availability

The datasets generated and/or analyzed during the current study are not publicly available due to the privacy policy (containing information that could compromise research participant privacy/consent), but are available from the corresponding author on reasonable request and with permission of parents of the involved children.
